# Abcb1 in Pigs: Molecular cloning, tissues distribution, functional analysis, and its effect on pharmacokinetics of enrofloxacin

**DOI:** 10.1038/srep32244

**Published:** 2016-08-30

**Authors:** Tingting Guo, Jinhu Huang, Hongyu Zhang, Lingling Dong, Dawei Guo, Li Guo, Fang He, Zohaib Ahmed Bhutto, Liping Wang

**Affiliations:** 1 College of Veterinary Medicine, Nanjing Agricultural University, Nanjing, 210095, PR China

## Abstract

P-glycoprotein (P-gp) is one of the best-known ATP-dependent efflux transporters, contributing to differences in pharmacokinetics and drug-drug interactions. Until now, studies on pig P-gp have been scarce. In our studies, the full-length porcine P-gp cDNA was cloned and expressed in a Madin-Darby Canine Kidney (MDCK) cell line. P-gp expression was then determined in tissues and its role in the pharmacokinetics of oral enrofloxacin in pigs was studied. The coding region of pig *Abcb1* gene was 3,861 bp, encoding 1,286 amino acid residues (Mw = 141,966). Phylogenetic analysis indicated a close evolutionary relationship between porcine P-gp and those of cow and sheep. Pig P-gp was successfully stably overexpressed in MDCK cells and had efflux activity for rhodamine 123, a substrate of P-gp. Tissue distribution analysis indicated that P-gp was highly expressed in brain capillaries, small intestine, and liver. In MDCK-pAbcb1 cells, enrofloxacin was transported by P-gp with net efflux ratio of 2.48 and the efflux function was blocked by P-gp inhibitor verapamil. High expression of P-gp in the small intestine could modify the pharmacokinetics of orally administrated enrofloxacin by increasing the Cmax, AUC and Ka, which was demonstrated using verapamil, an inhibitor of P-gp.

P-glycoprotein (P-gp, encoded by the *Abcb1* gene) is an important ATP-dependent efflux transporter, which was initially identified in Chinese hamster ovary cells in 1976^1^. P-gp has been documented to be highly expressed in the intestine, liver, and kidney of humans and rodents and as such has essential roles in bioavailability and drug-drug interaction of substrate xenobiotics^2^,^3^. P-gp has been shown to recognize structurally and pharmacologically diverse compounds, including drugs widely used in veterinary medicine (e.g. ivermectin, macrolides, and fluoroquinoles)^4^,^5^; thus, the relevance of P-gp in veterinary medicine and drug development is significant. However, little information is available for veterinarians on oral absorption, elimination and drug-drug interactions related to P-gp.

The complete cDNA of P-gp from human^6^, mouse^7^,^8^, rat^9^,^10^, ovine^11^, dog^12^, feline^13^ and the Salmon louse^14^ have been cloned. Pig is an important model species for veterinary medicine, and it is commonly used in toxicological and pharmacological studies. Until now, information about porcine P-gp is lacking, and expression and function of P-gp in pharmacokinetically important tissue is scarce. Here, full-length cDNA for porcine P-gp was cloned and a Madin-Darby Canine Kidney (MDCK) cell line that stably expressing pig P-gp was established. Its expression in tissues, as well as its participation in enrofloxacin pharmacokinetics, was assessed in pigs.

## Results

### Sequence analysis of porcine P-gp

**Figure 1:**
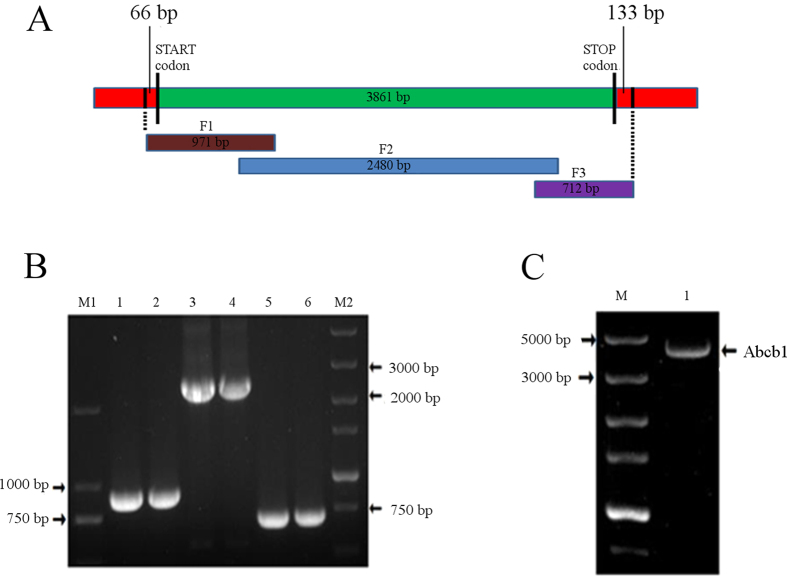
Summary of strategy to obtain full-length cDNA of porcine *Abcb1*. (**A**) Three overlapping fragments (F1, F2 and F3) were initially PCR amplified. Fragments F1, F2 and F3 were designed and sub-cloned into pMD-18-T vector (TaKaRa, Japan) for sequencing. (**B**) Three fragments of the porcine *Abcb1* amplified by PCR. M1: DNA Marker 2000, M2: DNA Marker 5000, Lanes 1, 2 - Fragment 1; Lanes 3, 4 - Fragment 2; Lanes 5, 6 - Fragment 3; The full-length gels are presented in Supplementary Figure 1. Gels were run under the same experimental conditions. (**C**) Full-length product amplified by fusion PCR using three fragments as templates. M: DNA Marker 5000, Lanes 1- Full-length cDNA of porcine *Abcb1*. Full-length gels appear in Supplementary Figure 1.

**Figure 2:**
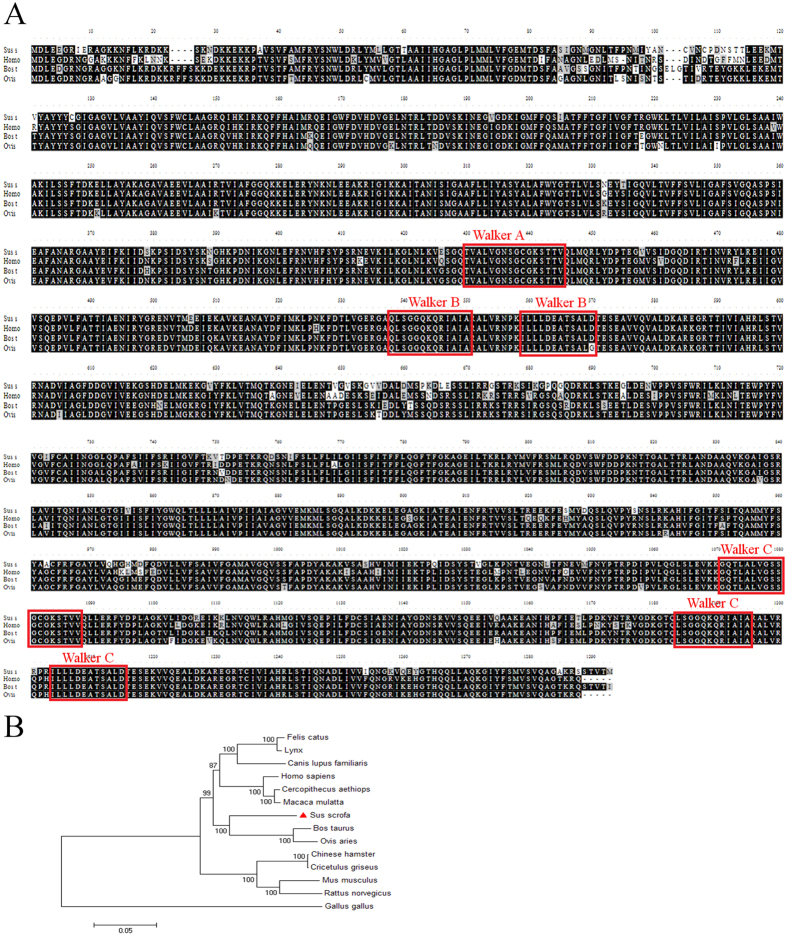
Bioinformatics analysis of porcine *Abcb1*. **(A**) Multiple sequence alignments of deduced amino acid sequence of pig *Abcb1* with other species. Alignments were performed with Bio Edit. Walker A-, Walker B-, and the C-motif are boxed in red, which are characteristic of ABC-transporters. GenBank ID: Homo sapiens NP_000918.2, Bos Taurus XP_590317.6, Ovis aries NP_001009790.1. (**B**) Phylogenetic tree of *Abcb1* coding sequences. Calculations were performed using the ClustalW algorithm and the evolutionary history was inferred using the Neighbor-Joining method in MEGA 4.0.

Based on sequence homology, three gene fragments of porcine *Abcb1*(971, 2480 and 712 bp) (Fig. 1A) were amplified (Fig. 1B) using primers depicted in Table 1. Fusion PCR was then performed to obtain full-length cDNA (Fig. 1C). The *Abcb1* gene was confirmed by DNA sequencing and BLASTN analysis at NCBI. The cDNA was 4,060 bp, consisting of a 5′-untranslated region of 66 bp, an uninterrupted open reading frame of 3,861 bp, and a 3′-untranslated region of 133 bp. The sequence was submitted to GenBank and assigned an accession number (GenBank ID: KP233220). The nucleotide sequence shared 89, 88, 89 and 82% identities with that of cow (XM590317.7), sheep (NM001009790.1), human (NM000927.4) and mouse (NM011075.2), respectively. The deduced amino acid sequence of porcine P-gp was 1,286 amino acid residues in length and the estimated molecular weight was 141.966 kDa and the theoretical isoelectric point was 8.99. The protein sequence of porcine P-gp shared 89, 88 and 87% identity to that of human (NP000918.2), cow (XP590317.6) and sheep (CAM33439.1), respectively (Fig. 2A). Unique to the sequences of P-gp in ruminants (sheep and cow) is a 4-amino acids deletion at position 22–25 of the amino acid sequence.

A phylogenetic tree was constructed using the neighbor-joining (NJ) method with a 1,000 bootstrap test based on the multiple alignments, which indicated that the selected 14 protein sequences of P-gp were clustered into two groups: avian and mammal. The porcine P-gp was located in vertebrates group and was closely related to cow and sheep, suggesting a close evolutionary relationship between them (Fig. 2B).

### Protein structural model of porcine P-gp

**Figure 3:**
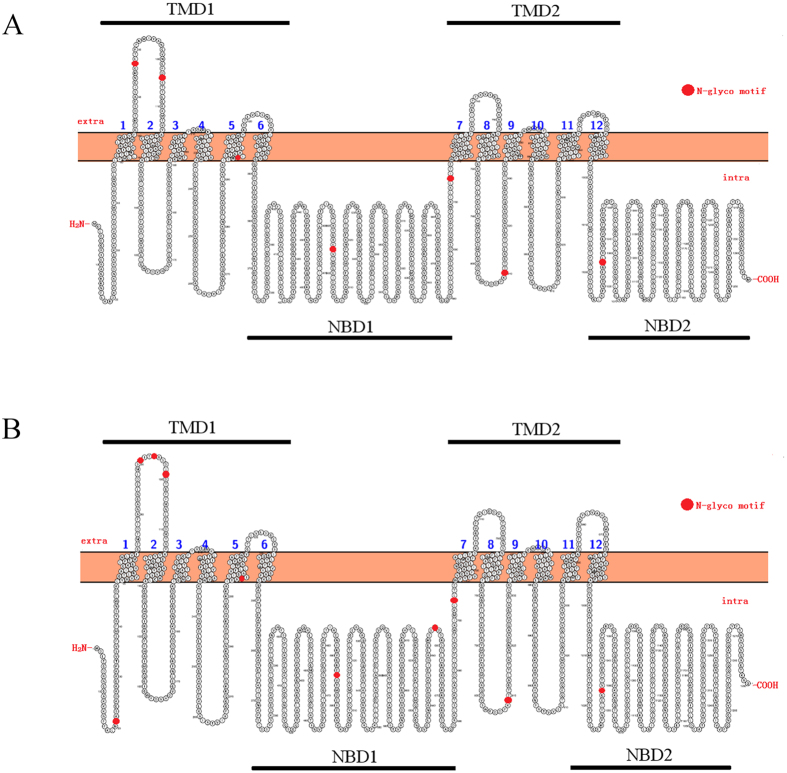
Secondary structure prediction of porcine P-gp (**A**) and human P-gp (**B**). Potential N-glycosylation sites of P-gp are colored with red (indicating position 87, 104, 297, 495, 705, 810 and 1035 in porcine P-gp sequence those differ from human P-gp).

**Figure 4:**
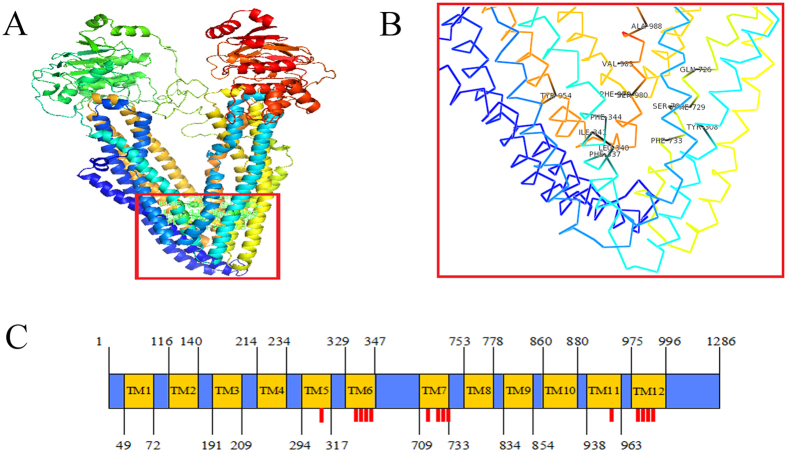
Protein 3D structure was modeled using I-TASSER online server. (**A**) Protein 3D structure with global view of ligand-binding domains. (**B**) Position of ligand-binding sites displayed using PyMOL molecular graphic software. (**C**) Locations of ligand-binding sites (red bar) in the transmembrane domains of porcine P-gp.

Porcine P-gp was predicted to possess 12 transmembrane helices with extracellular N- and C-termini (Fig. 3A) and comprised four structural domains: two cytoplasmic domains containing the nucleotide-binding domains (NBD) and another two hydrophobic transmembrane domains (TMD), which was similar to human P-gp (see Fig. 3). The potential putative N-glycosylation sites of porcine and human P-gp was marked in red (Fig. 3), indicating seven in the porcine P-gp, whereas there were ten in human P-gp. The tertiary structure of porcine P-gp contained 42 α-helices and 21 β-strands (Fig. 4A). Fourteen binding site residues were found to be located in TM5 (Tyr-308), TM6 (Phe-337, Leu-340, Ile-341, Phe-344), TM7 (Gln-726, Phe-729, Ser-730, Phe-733), TM11 (Tyr-954) and TM12 (Phe-979, Ser-980, Val-983, Ala -986) in the porcine P-gp (Fig. 4B,C).

### Establishment and characterization of an MDCK cell line stably-transfected with porcine *Abcb1*

**Figure 5:**
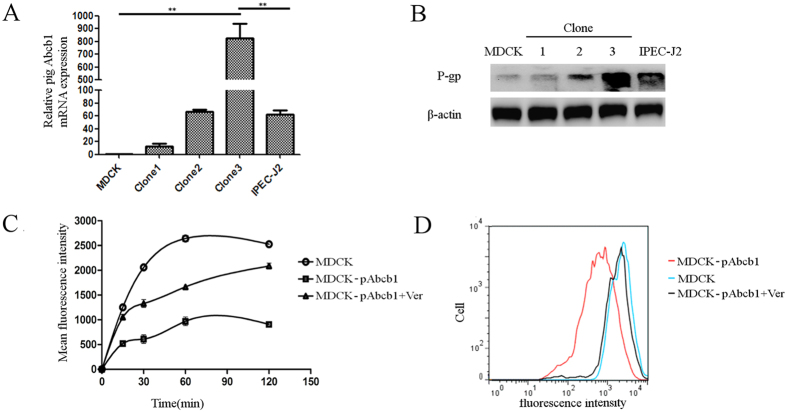
Establishment of a MDCK cell line that stably expressing porcine *Abcb1*. (**A**) qRT-PCR analysis of pig *Abcb1* mRNA levels in MDCK cells, IPEC-J2 cells and cells from three colonies of MDCK-pAbcb1. (**B**) Western blot of relative pig P-gp protein expression in MDCK, IPEC-J2 and cells from three colonies of MDCK-pAbcb1; assays run under the same experimental conditions and blots were cropped from original full-sized images (see Figure S2). Samples were derived from the same experiment and blots were processed simultaneously. (**C**) Accumulation of 5 μM Rho123 in MDCK and MDCK-pAbcb1 cells with/without verapamil (100 μM) during 2 h. Mean ± SEM; n = 3. (**D**) Rho123 accumulation at 2 h with flow cytometry. Histogram shows fluorescence (x-axis) representing Rho123 accumulation plotted as a function of the number of cells (y-axis) and is representative of three independent experiments.

The pig *Abcb1* gene was amplified using primers shown in Table 1 with *Xho*I and *Xba*I sites, respectively, and a recombinant pcDNA3.1-pAbcb1 plasmid was constructed. MDCK cells were transfected with pcDNA3.1-pAbcb1. A single cell colony resistant to G418 was selected as a stable transfectant (MDCK-pAbcb1). The presence of *Abcb1* was confirmed with qRT-PCR and western blot (Fig. 5A,B). The accumulation of Rho123 (substrate of human P-gp) was measured to investigate whether MDCK-pAbcb1 cell line has enhanced porcine P-gp protein function. Figure 5C,D show that MDCK-pAbcb1 cells accumulated less Rho123 than MDCK cells (control), indicating a significantly enhanced extruding capacity of Rho123 in MDCK-pAbcb1 cells (*p* < 0.01). Furthermore, verapamil (100 μM), an inhibitor of human P-gp, significantly (*p* < 0.01) increased Rho123 accumulation in MDCK-pAbcb1 cells, demonstrating that such an accumulation could be reversed by verapamil.

### Expression of P-gp in pigs

**Figure 6:**
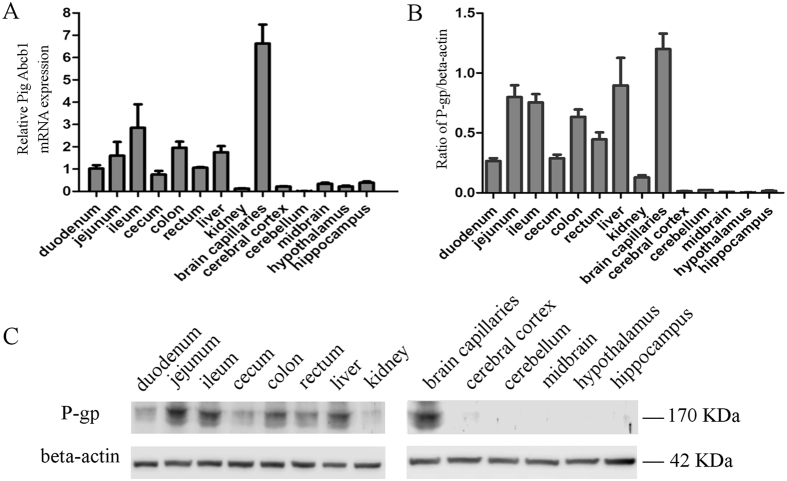
P-gp expression in different tissues. (**A**) Relative *Abcb1* mRNA levels; (**B**) Relative P-gp protein expression levels. Bars represent the mean ± SEM (n = 3). (**C**) Western blot of porcine P-gp protein; assays run under the same experimental conditions and blots were cropped from original full-sized images (see Figure S3). Samples were derived from the same experiment and blots were processed simultaneously.

Porcine P-gp mRNA and protein were analyzed by qRT-PCR and Western blot, respectively. As shown in Fig. 6A, *Abcb1* mRNA was detected in all tested tissues. In the CNS, the brain capillaries had the most *Abcb1* mRNA and the cerebellum had the least. *Abcb1* mRNA levels in the cerebral cortex, cerebellum, midbrain, hypothalamus and hippocampus was very low. In the peripheral tissues, *Abcb1* were highly expressed in the jejunum, ileum, colon and liver. *Abcb1* mRNA transcription in the ileum was greater than in the cecum and kidney (*p* *=* 0.005, *p* = 0.001) and significantly higher than that in the duodenum and rectum (*p* = 0.017, *p* *=* 0.014). *Abcb1* mRNA in the jejunum, colon and liver was significantly higher than that in the kidney (*p* *=* 0.046, *p* *=* 0.018, *p* *=* 0.030, respectively).

P-gp protein (shown in Fig. 6C) was ~170 kDa as detected in different pig tissues, indicating that antibody Mdr-1 directed against the human isoform could also recognize porcine P-gp. Protein expression in tissues appears in Fig. 6B. The most P-gp protein was in the brain capillaries and the least was observed in the hypothalamus. The P-gp protein level was correlated to its mRNA level (*r* = 0.863, *p* < 0.05).

### The effect of P-gp on *in vitro* transport and *in vivo* pharmacokinetics of enrofloxacin

Enrofloxacin was measured using HPLC, and the lowest limit of detection (LOD) of enrofloxacin was 0.02 μg/mL, based on a signal-to-noise ratio > 3. The lowest limit of quantification (LOQ) was 0.05 μg/mL with a signal-to-noise ratio > 10. The mean percent recovery of enrofloxacin exceeded 81%, and intra-assay and inter-assay reproducibility had a relative standard deviation (RSD) <11%. Assay linearity was good over 0.05–10 μg/mL with *r*^2^ = 0.9999.

MDCK and MDCK-pAbcb1 cells were grown to confluent monolayers on porous membrane filters, and vectorial transport of the enrofloxacin (12 μM) across the cells was determined. In the MDCK parental cell line, apically and basolaterally directed translocation of enrofloxacin were similar. However, in the pAbcb1 expressing MDCK cells, *P*_*app*_(BL-AP) was significantly higher than *P*_*app*_(AP-BL) (*P* < 0.01). The P-gp-mediated transport was completely inhibited by its inhibitor verapamil, resulting in a similar efflux ratio, compared to that of the MDCK parental cell line (Table 2). These results showed highly efficient transport of enrofloxacin by porcine P-gp.

**Figure 7:**
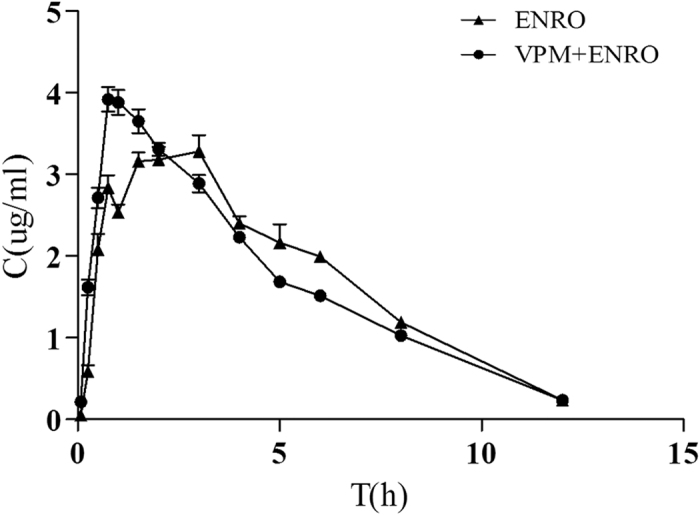
Mean plasma enrofloxacin in piglets after enrofloxacin administrated alone (10 mg/kg, po) and with verapamil (10 mg/kg, po). Each point represents the mean ± SEM of four piglets.

To further confirm whether P-gp affects the pharmacokinetics of enrofloxacin in pigs, verapamil (an inhibitor of P-gp) was administrated prior to enrofloxacin, and the plasma concentration-time curve and pharmacokinetics of enrofloxacin are summarized in Fig. 7 and Table 3, respectively. Compared with the control group, C_max_, AUC and Ka of enrofloxacin were increased, whereas T_peak_ and T_1/2ka_ of enrofloxacin were shortened in animals when co-administrated with verapamil. In summary, enrofloxacin pharmacokinetics was altered by P-gp inhibition, indicating that porcine P-gp can influence enrofloxacin behavior *in vivo*.

## Discussion

Oral drug administration is convenient intake route for humans, as well as for animals. The effects of P-gp on drug pharmacokinetics are important for the evaluation of the capacity of oral drug intake in pigs. The localization of P-gp in different tissues in pigs was studied in our previous work^15^; however, how P-gp functions in pigs is unclear. To address this deficit, we cloned full-length cDNA of porcine P-gp, and characterized the transport activity of P-gp by exogenously expressing the *Abcb1* gene in MDCK cells and measuring the porcine P-gp at the protein and transcriptional levels. Then, we assessed its role in the pharmacokinetics of oral enrofloxacin in pigs. This represents the first cloning of full-length porcine cDNA of P-gp.

The porcine P-gp shared high similarities with P-gps from cow, sheep, human and mouse, suggesting a similar physiological role in these animals. Drug-binding pockets are predicted to reside in transmembrane segments, which formed a translocation path for drug substrates as they exit the membranes^16^. The nucleotide binding domain may bind ATP or its analogs and can hydrolyze ATP^17^,^18^,^19^,^20^. There is “cross-talk” between nucleotide binding and transmembrane domains as evidenced by cross-linking studies^21^, and transmembrane domains of porcine P-gp were generated here with Protter protein display software. The prediction showed that porcine P-gp contains twelve transmembrane domains which was similar to human P-gp, but glycosylation site prediction revealed seven potential putative N-linked glycosylation sites on porcine P-gp, fewer than that in human P-gp. Some studies have shown that N-glycosylation can participate in many biological processes such as regulation of intracellular targeting, protein folding and maintenance of protein stability^22^,^23^. However, other studies have reported that N-linked glycosylation is not essential for protein expression, plasma membrane localization or overall function^24^,^25^. Further studies are necessary to clarify whether glycosylation affects the function of porcine P-gp.

To better understand how porcine P-gp transports various hydrophobic compounds, we predicted residues that participate in drug binding. Our results indicated that amino acids participating in substrate binding were located at TMD1 sites 5 and 6 and TMD2 sites 7, 11 and 12 of porcine P-gp, whereas previous studies have shown that human P-gp substrate binding sites were located in TMD1 sites 5 and 6 and TMD2 sites 11 and 12^26^. This may influence substrate types. Site-directed mutagenesis studies of *Abcb1* can be performed to prove whether these residues in porcine P-gp participate in ligand binding. Knowing substrate binding sites may enable development of drugs that bypass recognition of P-gp.

Human colorectal adenocarcinoma cell line (Caco-2) has been approved by the US Food and Drug Administration (FDA) for *in vitro* transport studies of P-gp^27^,^28^. However, pharmacokinetics might be different because of the specific functions of P-gp across different species^29^. An alternative approach was to establish a porcine P-gp expression system. The MDCK cell line is an approved model for overexpressing *Abcb1* originating from other species due to its negligible expression of endogenous transporters^30^,^31^. MDCK cell line overexpression porcine P-gp could be used to screen substrate drugs and potential inhibitors or inducers.

The efflux efficiency of P-gp substrate is determined by the P-gp number or density on the cell membrane^32^. Differences expression pattern of P-gp in tissues may influence therapeutic outcomes by affecting the accumulation of drugs in specific tissues. Transcription of *Abcb1* had been measured using RT-PCR in the liver and brain capillary endothelial cells (pBCECs)^33^ and in the kidneys^34^. We measured *Abcb1* mRNA level in the CNS, intestine, liver and kidneys. The data demonstrated that its expression in intestine was not uniform. In the peripheral tissues, the greatest expression was in the ileum. The porcine liver also expressed relatively high *Abcb1* mRNA, which was consistent with previous data from human and canines^35^,^36^. Renal *Abcb1* mRNA is lower in the pig, compared to that in humans^36^,^37^. P-gp in intestinal epithelial cells may prevent pharmacotherapeutic agents from entering the systemic circulation and therefore change their pharmacokinetics^38^. Hepatic metabolism is significant, and high expression level of P-gp is vital for biliary drug, toxicant, and metabolite excretion^39^. More *Abcb1* mRNA in the intestine and liver suggests that porcine P-gp may limit absorption and facilitate secretion of substrates into the intestines. In addition, we measured P-gp protein expression level in 60-day old piglets and found that this was coincident with mRNA levels, except for the ileum and jejunum, suggesting a tissue-specific post-transcriptional mode of regulation. Hence, RNA analysis of pig tissue samples should be done in conjunction with protein analysis, because RNA and protein may differ among certain organs.

Of note, the P-gp expression pattern in tissues affects pharmacokinetics of enrofloxacin, a fluoroquinolone (FQ) widely used in veterinary medicine either orally or parenterally. Sorgel’s group^40^ and others^41^,^42^,^43^ reported that the concentrations of ciprofloxacin and danofloxacin-mesylate in the gut lumen are higher than those in plasma. Other studies proved that ciprofloxacin and danofloxacin-mesylate were substrates of multiple human ABC transporters^44^,^45^,^46^, indicating that intestinal efflux is the underlying mechanism for danofloxacin-mesylate and ciprofloxacin secretion into the lumen. In this study, the net efflux ratio of enrofloxacin across MDCK-pAbcb1 cells was more than 2 and basolateral-to-apical transport of enrofloxacin could be counteracted by the P-gp inhibitor verapamil. Although the concentration of enrofloxacin was not measured in the luminal compartment, its concentration in plasma was increased when animals received verapamil. These results suggest that enrofloxacin might be a substrate of porcine P-gp, similar to the data obtained in chickens^47^. Initially, we treated piglets with the P-gp inhibitor PSC-833, but some animals began vomiting. Thus, PSC-833 was replaced with verapamil, which could inhibit P-gp function and be monitored in an MDCK-pAbcb1 cell line.

Our data also suggested that co-administration of P-gp inhibitors may be an alternative strategy to improve oral bioavailability and therapeutic efficacy of enrofloxacin when used to treat systemic bacterial infection in the swine industry. Three generations of P-gp inhibitors have been identified but none of them show improved therapeutic efficacy due to their broad activity and toxicity^48^. Berberine can significantly inhibit P-gp (data not shown) so it may be a potential P-gp inhibitor as it is not absorbed after oral administration and only inhibits P-gp in the gastrointestinal tract.

In conclusion, the complete cDNA of porcine P-gp was cloned for the first time. The protein structure was modeled and analyzed. The phylogenetic analysis on P-gp proteins from different species was performed, and its protein expression and mRNA levels in different tissues were studied. Also, the effect of P-gp on enrofloxacin transmembrane transport and *in vivo* pharmacokinetics was evaluated. More studies are required to fully elucidate the complex epigenetic regulation of porcine P-gp during development, tissue-specific expression patterns, and the contribution of epigenetics to species variability in drug disposition and therapeutic response.

## Materials and Methods

### Cells

MDCK (Madin-Darby canine kidney) and IPEC-J2 cells were obtained from Shanghai Institute of Cell Biology, the Chinese Academy of Sciences (Shanghai, China) and grown in Dulbecco’s modified Eagle’s medium (DMEM) supplemented with 10% fetal bovine serum (FBS, HyClone Laboratories Inc.). Cells were maintained at 37 °C in a humidified atmosphere of 5% CO_2_ and sub-cultured once 80% confluent.

### Animals

Sixty-day-old healthy crossbred pigs (large white x Landrace x Duroc, 20 ± 2 kg) were bought from Jiangsu Agricultural Academy and reared under standard conditions of light and temperature. Feed and water were provided *ad libitum* during the study. All pigs were fed according to the breeding standards of the Chinese Local Pigs and National Research Council (NRC). Animal use and handling protocols were approved by the regional Animal Ethics Committee and Nanjing Agricultural University. The protocol of the study was conducted in accordance with guidelines of the regional Animal Ethics Committee and Nanjing Agricultural University.

### Total RNA extraction and cDNA synthesis

All pigs were anesthetized and killed by decapitation. Samples of liver, kidney, intestines and brain tissues were taken immediately (within 5–10 min after death). Brain capillaries were isolated from ~10 g of fresh cerebral cortices according to published methods with minor modification^49^. In brief, meninges and choroid plexus from the brains were removed and cerebral matter was homogenized in 20 volumes of cold Ringer’s solution containing 10 mM HEPES (Santa Cruz, Heidelberg, Germany) at pH 7.4 in a glass homogenizer. Ten upwards and downwards strokes were applied during the homogenization. The homogenates were collected and filtered initially through a 150 μm nylon mesh, the filtrate was then re-filtered through a 60 μm nylon mesh. The brain capillaries are trapped on the 60 μm nylon meshes and were collected in the tube. All samples were frozen in liquid nitrogen and then stored at −80 °C until RNA and protein extraction.

Total RNA was extracted from tissues using TRIZOL (TaKaRa, Japan) following the manufacturer’s instructions. Quality and concentration were measured using a photometer (Eppendorf Biophotometer, Germany). RNA integrity was assessed with RNA electrophoresis. cDNA templates were synthesized from 1 μg of total RNA using a HISCRIPT 1^st^ strand cDNA synthesis kit (Vazyme, Nanjing, China).

### Cloning and sequencing of full-length cDNA of porcine *Abcb1*

PCR primers (Table 1) toward three overlapping cDNA fragments of porcine *Abcb1* (F1, F2, F3, shown in Fig. 1A) were designed based on sequence alignments of conserved regions from various species (Human, NM_000927.4; sheep, NM_001009790.1; dog, NM_001003215.1; mouse, NM_011075.2). Three overlapping fragments of porcine *Abcb1* were amplified by touchdown PCR using PrimeSTAR GXL DNA Polymerase (TaKaRa, Japan) under these conditions: 95 °C for 5 min, 36 cycles of 95 °C for 30 s, 65–55 °C for 30 s (initial annealing temperature of 65 °C was reduced by 2 °C after every six cycles to 55 °C for the final six cycles), 72 °C for 1 min, and an extra extension of 15 min at 72 °C. PCR products were verified using published methods^50^. After the three products were confirmed to be part of the *Abcb1* gene by sequencing, full-length cDNA was obtained from the three fragments using fusion PCR as follows: 95 °C for 5 min, 30 cycles of 95 °C for 30 s, 62.5 °C for 30 s, 72 °C for 1 min, and 72 °C for 15 min. Final PCR products were confirmed by sequencing and the sequence was submitted to GenBank to obtain an accession number.

### Bioinformatics analysis of porcine *Abcb1* and its protein structural model

cDNA sequence analysis was conducted using NCBI BLAST (http://www.ncbi.nlm.nih.gov/blast). Calculated molecular weights and predicted isoelectric points were obtained with the ExPASy online server (http://www.expasy.org/tools/). Multiple alignments of P-gp protein sequences were generated using the Bio Edit program. A phylogenetic analysis was used to determine relatedness of porcine P-gp to other mammalian P-gp sequences, and alignment data were imported into MEGA version 4.1. The phylogenetic tree was constructed using the neighbor-joining method with Poisson correction^51^. Bootstrap analysis was performed using 1,000 replicates.

The secondary structure of porcine and human P-gp was predicted and compared with Protter1.0 servers (http://wlab.ethz.ch/protter/start/)^52^ and 3D structures and precise substrate binding sites were predicted using I-TASSER (http://zhanglab.ccmb.med.umich.edu/)^53^.

### Establishment of an MDCK cell line stably-transfected with porcine *Abcb1*

Primers used to amplify pig *Abcb1* complementary DNAs (cDNAs) were designed based on our previously submitted sequence (GenBank ID: KP233220) containing an XhoI site within the sense primer and an Xba I site within the antisense primer (See Table 1 for primers). PCR amplifications were performed as previously described in the Methods. Pig *Abcb1* full cDNA was cloned into the plasmid pcDNA3.1 and transformed into *Escherichia coli* DH5α cells (Vazyme, China). Plasmid was purified using an Omega Endo-Free Plasmid Mini Kit and was sequenced using vector primers to confirm inserted genes (Invitrogen, Shanghai, China).

To generate cell lines stably expressing porcine P-gp, an MDCK cell line was transfected with pcDNA3.1-pAbcb1 plasmid using LipofectAMINE 2000 reagent (Invitrogen) according to the manufacturer’s instructions. Stable transfectants were selected with G418 (600 μg/mL, Gibco). When single-cell stable colonies resistant to G418 were observed, cells were sub-cultured again and distinct colonies were isolated using cloning cylinders. Cells were grown in the presence of G418 to maintain expression of transfected genes. MDCK -pAbcb1 cells were propagated and sub-cultured in the same medium with the addition of G418 (300 μg/mL). MDCK, IPEC-J2 and three colonies of MDCK -pAbcb1 were used to measure exogenous expression of porcine P-gp. qRT-PCR and western blot were used according to published methods^54^, with different primers (Table 1) and antibodies (Mdr-1(Santa Cruz, Heidelberg, Germany), dilution 1:200).

### Rhodamine 123 accumulation assay

MDCK and MDCK-pAbcb1 cells were seeded in six-well plates, and cells were washed with PBS and rhodamine 123 (Rho123; 5 μM) (Sigma-Aldrich, Castle Hill, Australia) was added in the presence and absence of verapamil (100 μM) to identify P-gp mediated Rho123 accumulation. To study the time-course of Rho123 accumulation, cells were harvested at 15, 30, 60 and 120 min after the addition of Rho123 and accumulation was measured using a FACS Calibur (BD Biosciences, Bedford, MA) with CellQuest Prosoftware. Data were collected for a minimum of 10,000 gated events per sample as geometric mean fluorescent intensity for all samples.

### Tissues distribution of P-gp (*Abcb1*) in pigs

Pigs (N = 6) were used to measure constitutive P-gp (*Abcb1*) expression in different tissues with real time quantitative PCR and western blot. *Abcb1* mRNA was measured with qRT-PCR and gene-specific primers for PCR were designed according to the cloned sequence of porcine *Abcb1* (KP233220 in GenBank). *GAPDH* was as internal control for each sample. Primer pairs for genes appear in Table 1. qRT-PCR was performed on a CFX96 Real-Time PCR Detection System (Bio-Rad, USA), and SYBR Green was the dye (Toyobo, Japan). mRNA was measured using 12.5 μL SYBR Green real-time PCR Master Mix in a 25 μL volume with 2 μL cDNA. Each run consisted of an initial 1 min activation cycle at 95 °C, followed by 40 cycles of denaturation for 20 s at 95 °C, annealing for 30 s at 60 °C, and elongation for 31 s at 72 °C. Uniform amplification of products was analyzed according to melting curves of the amplified products. *Abcb1* mRNA relative expression in different tissues was measured using the 2^−ΔΔCt^ method^55^.

P-gp relative expression was measured with standard western blot with minor modifications^50^. Tissue protein was extracted using a membrane and cytosol protein extraction kit (Beyotime, Haimen, China). Briefly, equal amounts of membrane proteins (5 μg/lane) were fractionated with SDS-PAGE and then transferred onto PVDF membranes (BIO-RAD, USA). Membranes were blocked with 5% bovine serum albumin and incubated with primary antibody at the appropriate dilution (Mdr-1, Santa Cruz, Germany, 1:200; β-actin, TransGen, China, 1:5,000). Membranes were incubated overnight at 4 °C, followed washing three times with PBST for 15 min. Thereafter, membranes were incubated with horseradish peroxidase-labeled goat anti-mouse IgG secondary antibody (1:5,000, Boster, Wuhan, China) for 1 h at 37 °C, then washed three times with PBST for 15 min and two times with PBS for 10 min. Interactive bands were visualized with ECL (Vazyme, China) and scanned with a Tanon 5200 chemiluminescent imaging system (Tanon, China). Relative protein was quantified by calculating densitometric values of target bands using Image J software.

### Experimental design of enrofloxacin bidirectional transport assay and pharmacokinetic studies in pigs

Transport assays were carried out as described^45^ with minor modifications. MDCK and MDCK-pAbcb1 with a density of 6.6 × 10^4^ cells/insert were seeded on a polyethylene terephthalate membrane insert (Millicell cell culture inserts, 1.0 μm pore size, 6.5 mm diameter) in 24-well culture plates. Cell monolayers with TEER values > 150 Ω.cm^2^ were used in the study^56^. Two hours before the experiment, medium in both apical and basolateral sides of the monolayer was replaced by HBSS transport buffer, either with or without 100 μM verapamil. The experiment was started (t = 0) by replacing the medium in either the apical (AP) or basolateral (BL) compartment with fresh HBSS buffer containing 12 μM enrofloxacin in the presence or absence of 100 μM verapamil. Transwells were shaken gently at 50 rpm. At 120 min, 0.20 ml samples were withdrawn from the receiver chambers. Samples were stored at −20 °C. All experiments were conducted in triplicate.

To evaluate the effect of P-gp on the pharmacokinetics of enrofloxacin, animals (N = 8 total) were randomized to 2 groups. Group 1 animals received enrofloxacin (10 mg/kg, po), and each animal in group 2 was pre-treated with verapamil (10 mg/kg, po) and then enrofloxacin (10 mg/kg, po). Blood samples were collected from the jugular vein 20 min before and at 0.083, 0.25, 0.5, 0.75, 1, 1.5, 2, 3, 4, 5, 6, 8, 12 and 24 h after enrofloxacin administration. Samples were centrifuged at 1,500 × g for 10 min and plasma was harvested and aliquoted for storage at −80 °C before HPLC analysis.

### HPLC analysis of enrofloxacin

HPLC analysis of enrofloxacin was performed as published with minor modification^47^,^57^. Concentrations of enrofloxacin were measured using an Agilent 1200 HPLC system. In brief, plasma was thawed at room temperature and centrifuged at 2,000 × g for 5 min. The supernatant (0.5 mL) was mixed with acetonitrile and separated into organic and water phases by centrifugation. The organic phase was evaporated to dryness under a nitrogen stream and residue was resuspended with mobile phase solution. The samples from the transport assays were directly evaporated to dryness under a nitrogen stream and 40 μL of the mobile phase solution was used to concentrate the samples for 5 times. Twenty microliters of the mixture were injected into the HPLC column (Kromasil C18 columns, 5 mm particle size, 250 × 4.6 mm). The mobile phase composition was 0.1 M phosphoric acid (adjusted pH to 3.5 with triethylamine)/acetonitrile (84:16, v/v) and the flow rate of mobile phase was set to 0.85 mL/min. UV absorbance was measured at 278 nm. Assay validation including recovery rate, inter- and intra-assay precision, accuracy and assay linearity of enrofloxacin were assessed using published methods^58^.

### *P*_*app*_ Calculation and Pharmacokinetic analysis

Apparent permeability coefficients (*P*_*app*_) were calculated using the following equation: *P*_app_ = (dQ/dt)/(A × C_0_), where A is the area of filter membrane, C_0_ is the initial concentration of the test drug, dQ is the amount of transported drug, and dt is time elapsed. The efflux ratio (ER) was calculated from (*P*_app_ B → A)/(*P*_app_ A → B)^59^. Where *P*_*app*_ B → A and *P*_*app*_ A → B are BL to AP and AP to BL apparent permeability coefficients, respectively. The net efflux ratio was calculated using the following equation: Net efflux ratio = efflux ratio in MDCK-pAbcb1/efflux ratio in MDCK.

Pharmacokinetics were calculated for individual data using 3p97 practical pharmacokinetic software (Version97, Chinese Pharmacologic Association, Beijing, China). The best fit compartment model was assessed according to Akaike’s information criterion.

### Statistical analysis

All data were analyzed for statistical significance using SPSS Statistics 17.0 (version 17.0, SPSS Inc., Chicago, IL, USA). The differences between means were considered significant at *p* < 0.05 and very significant at *p* < 0.01. Data were shown as means ± SEM.

## Additional Information

**How to cite this article**: Guo, T. *et al.* Abcb1 in Pigs: Molecular cloning, tissues distribution, functional analysis, and its effect on pharmacokinetics of enrofloxacin. *Sci. Rep.* **6**, 32244; doi: 10.1038/srep32244 (2016).

## Supplementary Material

Supplementary Information

## Figures and Tables

**Table 1:** Primers used in this study.

Primers used for Abcb1 cloning		
Fragments of Abcb1	Sequences (5′-3′)	Length (bp)
F1	F: GTCTGCCTCTCTTCTTCCAAAAATC	971
	R: GCACCAATAGAAATGTTGGCTGTAATAGC	
F2	F: GAAGAAGCCAAAAGGATTGGAATAAAGAAA	2,480
	R: CACTGGACGTTCAGTTTTTTAATCTCCC	
F3	F: GTGCTAATTGACGGCAGGGAGATTA	712
	R: GTGGAATGATCTTCAATGGTAGCAGG	
Primers used for real-time PCR		
Genes	Sequences (5′-3′)	Length (bp)
Abcb1	F: AGTCTAATAAGAAGAGGAT	145
	R: GCCATTCAGTTATATTCA	
Gapdh	F: GAAGGTCGGAGTGAACGGAT	149
	R: CATGGGTAGAATCATACTGGAACA	
Primers used for plasmid construction		
Genes	Sequences (5′-3′)	Length(bp)
Abcb1	F: GACCGctcgagGCCACCATGGATCTTGAAGAAGGCCG	3,861
	R: GCAACtctagaTCACATGGTCACAGTTGATGAG	

**Table 2:** Bidirectional transport assay in MDCK and MDCK-pAbcb1 cells (mean ± SEM, n = 3).

	*P*_app(AP-BL)_ (×10^−6^ cm/s)	*P*_app(BL-AP)_ (×10^−6^ cm/s)	Efflux ratio	Net efflux ratio
ENRO				
MDCK	12.35 ± 0.59	12.94 ± 1.65	1.04 ± 0.11	—
MDCK-pAbcb1	7.78 ± 1.28	19.89 ± 1.57^##^	2.58 ± 0.25^##^	2.48
ENRO + VER				
MDCK	11.66 ± 0.99	12.18 ± 1.23	1.05 ± 0.15	—
MDCK-pAbcb1	11.79 ± 0.92	14.47 ± 1.22	1.24 ± 0.19**	1.19

Statistical significance was analyzed by Student’s t-test. ^##^*P* < 0.01 significant difference of enrofloxacin transport in MDCK and MDCK-pAbcb1 cells; **P < 0.01 significant difference of enrofloxacin transport in presence/absence of verapamil.

**Table 3:** Oral enrofloxacin in healthy pigs with/without verapamil (mean ± SEM, n = 4).

Pharmacokinetics	ENRO	ENRO + VER
Ke (h^−1^)	0.28 ± 0.03	0.25 ± 0.02
Ka (h^−1^)	0.82 ± 0.11	2.08 ± 0.11**
T_1/2ka_ (h)	0.88 ± 0.20	0.34 ± 0.02**
T_1/2ke_ (h)	2.50 ± 0.23	2.85 ± 0.22
T_peak_ (h)	2.01 ± 0.09	1.17 ± 0.05**
C_max_ (μg/mL)	3.39 ± 0.12	3.85 ± 0.15*
AUC (μg/mL)·h	13.57 ± 0.96	16.50 ± 0.44*
CL/F (mg/kg/h)/(μg/mL)	0.47 ± 0.01	0.48 ± 0.01
V/F (mg/kg)/(μg/mL)	1.69 ± 0.18	1.96 ± 0.11

*P < 0.05, **P < 0.01 significant difference for enrofloxacin in presence/absence of verapamil in healthy pigs. Ke, elimination rate constant; Ka, absorption rate constant; T_1/2ka_, the absorption half-life; T_1/2ke_, the elimination half-life; T_peak_, the time to reach peak concentration; C_max_, the peak concentration; AUC, the area under the plasma concentration-time curve; CL/F, Clearance/F, where F is the fraction of dose absorbed; V/F, volume of distribution/F, where F is the fraction of dose absorbed.
