# Supplementary material for: Abcb1 in Pigs: Molecular cloning, tissues distribution, functional analysis, and its effect on pharmacokinetics of enrofloxacin
Source: Sci Rep. 2016 Aug 30;6:32244. doi: 10.1038/srep32244 (PMC5004175; doi:10.1038/srep32244)

**Abcb1 in Pigs: Molecular cloning, tissues distribution, functional analysis, and its effect on pharmacokinetics of enrofloxacin**

Authors and Affiliations

Tingting Guo, Jinhu Huang, Hongyu Zhang, Lingling Dong, Dawei Guo, Li Guo, Fang He, Zohaib Ahmed Bhutto, Liping Wang *

1. College of Veterinary Medicine, Nanjing Agricultural University, Nanjing, 210095, PR China

*Corresponding author: Dr. Wang Liping, College of Veterinary Medicine, Nanjing Agricultural University, Nanjing, 210095, PR China. Phone: +86-025-84395573; Fax: +86-025-84398669; Email: [wlp71@163.com](mailto:wlp71@163.com)

Supplementary Figure S1. PCR products of porcine Abcb1. M1：DNA Marker 2000，M2：DNA Marker 5000, Lanes 1, 2 - Fragment 1; Lanes 3, 4 - Fragment 2; Lanes 5, 6 - Fragment 3; M2：DNA Marker 5000，Lanes 7- Full-length cDNA of porcine Abcb1.


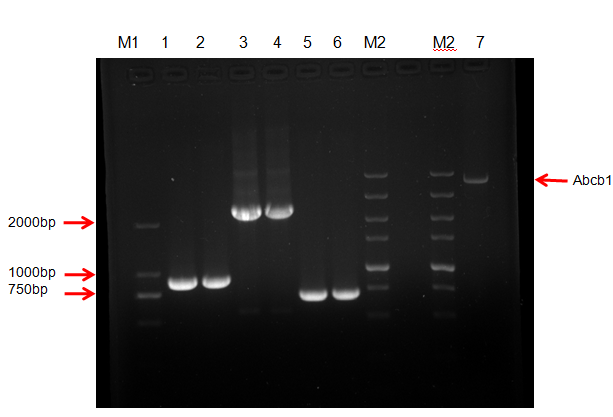
Supplementary Figure S2. Western blot of relative pig P-gp protein in MDCK, IPEC-J2 and cells from three colonies of MDCK-pAbcb1. Full immunoblots corresponding to the cropped versions shown in Fig.5B. The samples were derived from the same experiment and that blots were processed in parallel.







Supplementary Figure S3. Western blot of porcine P-gp and beta-actin. Full immunoblots corresponding to the cropped versions shown in Fig.6C. The samples were derived from the same experiment and that blots were processed in parallel.


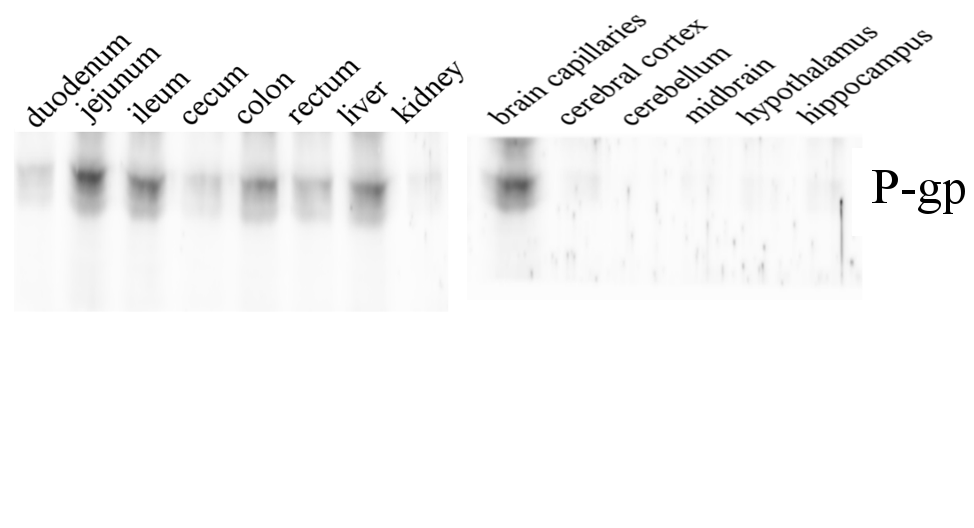

Supplement: Supplementary Information [file srep32244-s1.docx]
